# Prolonged prone position in pregnant woman with COVID-19 pneumonia

**DOI:** 10.1186/s44158-022-00044-9

**Published:** 2022-04-12

**Authors:** Federico Linassi, Matteo Campagnolo, Enrico Busato, Valentina Ortolani, Mario Peta

**Affiliations:** 1 Department of Anesthesiology and Critical Care, Treviso Regional Hospital AULSS 2 Marca Trevigiana Piazzale Ospedale 1, 31100 Treviso, Italy; 2 Department of Pharmaceutical and Pharmacological Sciences, Università degli Studi di Padova, via Marzolo 5, 35131 Padova, Italy; 3 Department of Gynaecology and Obstetrics, Treviso Regional Hospital AULSS 2 Marca Trevigiana Piazzale Ospedale 1, 31100 Treviso, Italy

**Keywords:** COVID-19, Prone position, ARDS

## Abstract

The manuscript describes a case report of 2 prolonged prone position cycles (72 h each) of a coronavirus disease 2019 pneumonia in an intubated pregnant woman (at 22 weeks of gestational age), being successfully discharged from intensive care unit after 20 days. There were no signs of fetal sufferance at daily obstetric monitoring during prone position, and the fetus was born fully vital and without consequences.

At our knowledge, this is the first case of prolonged prone position in a pregnant woman, and we feel that our manuscript could be a valuable contribution to the literature and help intensivists in providing intensive care in these patients, confirming that prone position seems to be a valid therapeutic choice, limiting maternal and fetal hypoxia, and reducing their morbidity, even if the oculate risk/benefit should be performed. Further studies are however necessary to increase the knowledge and the good management of COVID-19 in pregnancy.

To the Editor,

From the beginning of COVID-19 (coronavirus disease-19), we gained more data on disease severity, course, and treatment of patients affected by this disease. However, limited data are available about intensive care unit (ICU) conduct particularly on the application of prolonged prone position ventilation [1] in severe acute respiratory distress syndrome (ARDS) pregnant patients [2, 3].

Here we report a case of a 40-year-old Moroccan woman, at the 22th week of gestational age, with only hypothyroidism in anamnesis. She was admitted to our Emergency department for COVID-19 bilateral pneumonia, after 10 days from the beginning of symptoms (fever and cough), and after 2 days of hospitalization in the medical department, where respiratory dynamic and exchanges worsened, although incremental FiO_2_). At ICU admission (day 0, D0) she had tachypnea and severe hypoxia (PaO_2_/FiO_2_ of 60 in high flow nasal cannula (HFNC)). She was initially treated with steroids (dexamethasone 6 mg daily for 7 days), prophylactic Low Molecular Weight Heparin and non-invasive ventilation with full-face mask, reaching a PaO_2_/FiO_2_ of 116. Lung ultrasounds were provided daily to the patient [4], documenting multiple subpleural anterior, posterior and inferior lung consolidations, without pneumothorax or pleural effusion occurence. Her first chest radiography is reported in Fig. 1A. But on D2, for clinical worsening and an increasing severe dyspnea and after a collegial discussion involving also our gynecological department, she was intubated and pronated, undergoing a first long-prone position cycle (72 h, according to our hospital protocol), limiting abdominal and pelvic compression with proper positioning and cushions. The patient was firstly treated with prophylactic dosage of ceftriaxone (5 days), then with vancomycin for increase in inflammation indices and an isolation of a Staphylococcus Aureus in a Braoncoaspiratus. Best PEEP was determined every 12 h, determining it according to our protocols [5]. During the first cycle of the prone position she initially required high Positive End-Expiratory Pressure (PEEP of 14 cmH_2_O, after 36 h reduced to 12 cmH_2_O), with a driving pressure of 12 cmH_2_O, after 48 h (D4) reduced to 10 cmH_2_O, and a respiratory rate of 16, on D4 reduced to 14, reaching a PaO_2_/FiO_2_ of 234 when she was supinated (after 72 h, on D5); However during the first 24 h of supination (still curarized, on D6) her respiratory parameters worsened, so she underwent a second cycle of prolonged prone position, requiring similar ventilation settings. After another 72 h, she reached a P/F of 290, so she was finally supinated at D9. PaCO_2_ was always maintained at a range 35–45 cmH_2_O. Daily intra-abdominal pressure (IAP) was also performed, starting from an IAP of 10 cmH_2_O (in the supine position) and reaching a maximum of 13 during prone position. However, since the best PEEP titration in patients with increased IAP is still debated [6], we titrated it on the sole base of ARDS protocols [5]. No maternal complications due to prolonged prone position were observed, and daily and obstetric monitoring of fetal well-being with cardiotocography and weekly ultrasounds and maternal uterine artery Doppler flow velocimetry were performed; Even if an umbilical arterial systolic/diastolic ratio decrease can be expected in the prone position [7], we did not observe them, maybe because obstetric evaluations were performed in right lateral decubitus when the patient was in the prone position. However, no fetal sufferance signs were shown.

**Fig. 1:**
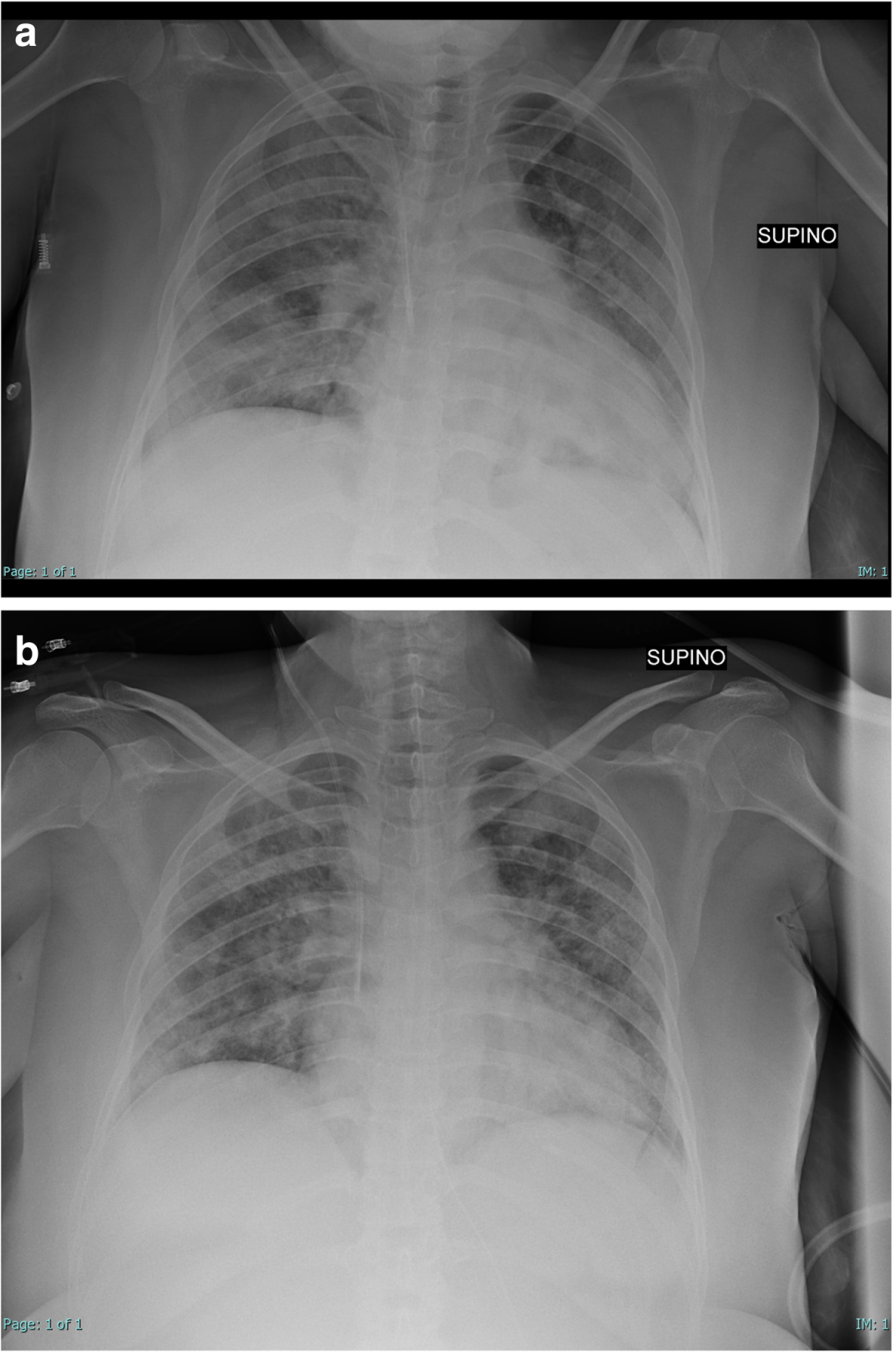
**a** Chest radiography of the pregnant woman before intubation, at ICU admission. **b** Chest radiography of the pregnant woman after extubation (15th day)

She was finally extubated on D15 (Fig. 1B), supported with HFNC until discharge in the medical department (D20), and finally discharged from the hospital on D26. All of the obstetric monitoring showed fetal activity, compatible with the maternal sedation status.

This woman underwent a cesarean section at 38 weeks and 6 days of pregnancy, with a female newborn who was fully vital (5 min—Apgar score of 10).

According to our case, intubation and prolonged prone position seems to be compatible with fetal survival, at least within the second quarter of pregnancy;

Intubation and prolonged prone position have to be considered as the “last chance” for pregnant women, performing an oculate risk/benefit evaluation; however, we can confirm their rule in limiting maternal and fetal hypoxia, and in reducing their morbidity [8, 9].

Further studies are however necessary to increase the knowledge and the good management of COVID-19 in pregnancy.

## Data Availability

Authors declare that the datasets used and/or analyzed during the current study are available from the corresponding author on reasonable request.
